# Nomogram for predicting invasive lung adenocarcinoma in small solitary pulmonary nodules

**DOI:** 10.3389/fonc.2024.1334504

**Published:** 2024-07-01

**Authors:** Mengchao Xue, Rongyang Li, Junjie Liu, Ming Lu, Zhenyi Li, Huiying Zhang, Hui Tian

**Affiliations:** Department of Thoracic Surgery, Qilu Hospital of Shandong University, Jinan, China

**Keywords:** solitary pulmonary nodules, diagnosis, prediction, logical model, invasive pulmonary adenocarcinoma

## Abstract

**Background:**

This study aimed to construct a clinical prediction model and nomogram to differentiate invasive from non-invasive pulmonary adenocarcinoma in solitary pulmonary nodules (SPNs).

**Method:**

We analyzed computed tomography and clinical features as well as preoperative biomarkers in 1,106 patients with SPN who underwent pulmonary resection with definite pathology at Qilu Hospital of Shandong University between January 2020 and December 2021. Clinical parameters and imaging characteristics were analyzed using univariate and multivariate logistic regression analyses. Predictive models and nomograms were developed and their recognition abilities were evaluated using receiver operating characteristic (ROC) curves. The clinical utility of the nomogram was evaluated using decision curve analysis (DCA).

**Result:**

The final regression analysis selected age, carcinoembryonic antigen, bronchus sign, lobulation, pleural adhesion, maximum diameter, and the consolidation-to-tumor ratio as associated factors. The areas under the ROC curves were 0.844 (95% confidence interval [CI], 0.817–0.871) and 0.812 (95% CI, 0.766–0.857) for patients in the training and validation cohorts, respectively. The predictive model calibration curve revealed good calibration for both cohorts. The DCA results confirmed that the clinical prediction model was useful in clinical practice. Bias-corrected C-indices for the training and validation cohorts were 0.844 and 0.814, respectively.

**Conclusion:**

Our predictive model and nomogram might be useful for guiding clinical decisions regarding personalized surgical intervention and treatment options.

## 1 Introduction

Solitary pulmonary nodules (SPNs) are single well-defined imaging opacities ≤ 30 mm in diameter surrounded by lung parenchyma without pulmonary atelectasis, mediastinal lymph node enlargement, or pleural effusion (1). Solitary pulmonary nodules ≤ 20 mm are defined as small (2). The present dominant technique for lung cancer screening is high-resolution computed tomography (CT), which has substantially increased detection rates of isolated, particularly, of small SPNs (3–5). In clinical practice, overall survival is similar for lesions ≤2 cm removed by sublobar resection and lobectomy. The choice of sublobar or lobectomy is debatable. Adenocarcinoma is the most prevalent histological type of lung cancer and its incidence has recently increased (6–12). The World Health Organization (WHO) classification of lung tumors (2021) has categorized lung adenocarcinomas as preinvasive lesions that include atypical adenomatous hyperplasia (AAH), adenocarcinoma *in situ* (AIS), minimally invasive (MIA) and invasive (IAC) adenocarcinoma (13).

A recently proposed pathological classification is that lung adenocarcinoma should be categorized as pre-invasive pulmonary adenocarcinoma (IPA) and IPA. Pre-IPA lesions comprise AAH, AIS, and MIA (14, 15). Clinical treatment tends to differ between pre-IPA and IPA; sublobar resection might be reasonable for pre-IPA lesions because the 5-year survival rate after complete resection is ~ 100%, whereas standard lobectomy and lymph node dissection coverage might be suitable for IPA (15, 16). However, to distinguish pre-IPA from IPA lesions is difficult in the absence of complete preoperative histological sampling, which limits optimal treatment planning (17). Therefore, an effective preoperative risk prediction model is needed to predict IPA risk.

Numerous prediction models, including the most well-known Mayo model, the Brock University model, the Peking University People’s (PKUPH) model, the VA model, and others, have been developed to date for SPN diagnosis. Over 80% of these models have demonstrated diagnostic accuracy. Every model, in the meantime, has flaws of its own and requires more optimization.

A nomogram is a reliable tool for creating simple visual graphs of statistical predictive models to quantify the risk of clinical events such as cancer (18, 19). The high incidence of lung adenocarcinoma prompted us to develop a risk prediction model to differentiate IPA from pre-IPA in patients with isolated lung nodules and to establish a nomogram combining CT and clinical features to determine IPA risk in patients with SPNs to support clinicians’ treatment recommendations.

## 2 Materials and methods

### 2.1 Patient selection

The Ethics Committee of Qilu Hospital, Shandong University approved this single-center study (registration number: KYLL-202008–023-1) and waived the need for written informed consent due to its retrospective design. All procedures complied with the principles enshrined in the Declaration of Helsinki (2013 amendment).

This study included patients with small SPNs with clear pathology who underwent minimally invasive pulmonary resection between January 2020 and December 2021 at the Department of Thoracic Surgery, Qilu Hospital, Shandong University. Inclusion criteria comprised: a single intrapulmonary nodule suggested by chest CT within 1 month before surgery, SPN diameter ≤ 20 mm, absent pulmonary atelectasis and active lung inflammation, surgical resection to obtain definitive pathological findings. Asymptomatic at diagnosis, and no preoperative treatment. Exclusion criteria comprised age < 18 years, open thoracic surgery, incomplete perioperative data, history of malignant disease within 5 years, and metastatic tumors. All those who met the criteria were randomly assigned using a random split sample method to training and validation cohorts in a 7:3 ratio to respectively develop and verify the performance of a prediction nomogram.

### 2.2 Data collection and variable definitions

We downloaded the following information about the patients from the Qilu Hospital database: demographic data: sex, age, smoking history, body mass index (BMI), preoperative comorbidities (hypertension, diabetes, and chronic obstructive pulmonary disease); preoperative assessment outcomes (American Society of Anesthesiologists scores, % predicted forced expiratory volume in one second, % predicted maximal voluntary ventilation), laboratory blood findings [blood type, blood sugar, serum complement C1q, serum 5’-nucleotidase, lactate dehydrogenase, serum amyloid, albumin, neutrophils, lymphocytes, basophils, eosinophils, monocytes, erythrocytes, hemoglobin, platelets, prognostic nutritional index (PNI), neutrophil-to-lymphocyte ratio (NLR), platelet-to-lymphocyte ratio (PLR), monocyte-to-lymphocyte ratio (MLR), derived neutrophil-to-lymphocyte ratio (dNLR), neutrophil-to-lymphocyte and platelet ratio (NLPR), aggregate index of systemic inflammation (AISI), systemic inflammatory response syndrome (SIRS), systemic inflammation index (SII), pan-immune-inflammation value (PIV)]; lung cancer tumor markers (cytokeratin 19-fragments, squamous cell carcinoma antigen, pro-gastrin-releasing peptide, carcinoembryonic antigen (CEA), carcinoma antigen 125, and neuron-specific enolase); imaging features (shape regular or irregular), location (central or peripheral), spiculation (sunburst appearance), calcification, pleural adhesions, lobulation, cavitation, vascular penetration, lymph node enlargement, bronchus and, pleural effusion signs, maximum tumor diameter, consolidation-to-tumor ratio (CTR) and pathology data: postoperative pathological malignant SPN ≤ 2 cm).

AISI, [(neutrophils × monocytes × platelets)/lymphocytes].
dNLR, [neutrophils/(leukocytes - neutrophils)].
MLR, monocytes/lymphocytes.
NLPR, [Neutrophils/(lymphocytes × platelets)].
NLR, neutrophils/lymphocytes.
PIV, [(neutrophils × platelets × monocytes)/lymphocytes].
PLR, platelets/lymphocytes.
PNI, serum albumin (g/L) + 5×total lymphocyte count (×109/L).
SII, [(neutrophils x platelets)/lymphocytes)].
SIRI, [(neutrophils × monocytes)/lymphocytes)].

The study’s blood collection time was standardised, and on the morning of the second hospital day, all patients had their blood drawn while fasting and in a peaceful state. Results from blood tests were obtained for each patient no later than one week before to surgery.

All scans were performed with Iopromide injection 300 contrast enhancement from the base to the apex of the lung using either a 64-slice multi-detector CT (Aquilion 64; Toshiba Medical Systems) or a 16-slice multi-detector CT (Somatom Definition AS, Siemens Healthcare, Erlangen, Germany). The patients were lying supine when the scans were obtained at the conclusion of inspiration. The scanning parameters were 50 mA, 1 mm collimation, 1.5:1 pitch, and 120 kVp. With filtered back projection, a 2 mm slice thickness, and a 2 mm increment, the data were recreated using a smooth convolution kernel (Siemens B30f or Toshiba FC02). Computed tomography images of the entire chest during deep inspiration and breath-holding were acquired from supine patients. Two radiologists with > 5 years of experience in chest radiology independently measured each imaging feature, and another with >20 years of experience in chest radiology reassessed discrepancies. Disagreements were resolved by consensus. Centrality was defined nodules in the bronchi, lobar bronchi, or lung segmental bronchi. Peripheral location was defined as nodules found below the tertiary bronchus. Spiculation was defined as the spread of strands from the nodal margins into the lung parenchyma without contacting the pleural surface. Calcification signs on CT images were defined as stratification, central nodule, bronchi, diffusion, or popcorn. Cavitation signs were defined as gas-filled spaces that are considered as transparent or low-attenuation regions. Vascular penetration was assumed when a pulmonary artery crossed a node. Pleural adhesion was defined as linear attenuation of the pleura or a major or minor fissure from the SPN. The bronchial sign indicated direct bronchial involvement of the nodules. Lobulation was defined as a wavy or fan-shaped portion of the lesion surface, with strands extending from the nodal margins into the lung parenchyma. Pleural effusion was defined as blunting of the rib-diaphragm angle. Mediastinal lymph node enlargement was noted. The CTR is the ratio of the diameter of the solid component of a lung nodule to its maximum diameter.

All pathological specimens were fixed in formalin, stained with hematoxylin and eosin, and histologically evaluated by two experienced lung pathologists using a light microscope. All specimens were categorized according to the International Association for the Study of Lung Cancer/American Thoracic Society/European Respiratory Society classification of lung adenocarcinoma (20).

We assigned patients with SPN diameters ≤ 2 cm to pre-IPA and IPA groups. The pre-IPA group included patients with AAH, AIS, MIA, and benign lesions.

### 2.3 Statistical analysis

All data were statistically analyzed using SPSS (version 26.0; IBM Corp., Armonk, NY, USA). Normally distributed continuous variables are expressed as means ± standard deviation (SD) and compared using Student t-tests. Non-normally distributed continuous variables are expressed as medians with interquartile ranges (IQRs) and two groups were compared using Mann-Whitney U tests. Categorical variables were compared using Pearson chi-square or Fisher exact tests. The statistical significance of differences was defined at P < 0.05. All risk factors affecting the probability of IPA in the training cohort were evaluated using univariate analysis, then all those with p < 0.05 in were included in multivariate logistic regression analysis using R statistical software (Windows version 4.2.1, http://www.r-project.org/). A predictive model for SPN was constructed based on the results of multiple logistic regression analyses. The area under the receiver operating characteristic (ROC) curves (AUC) was determined. Scores for each variable were calculated using a regression model, and the predictive probability of IPA was derived by adding the scores for each variable. Nomograms were built and calibration curves were generated using the regression modeling strategies (rms) package in R. The ROC curves were plotted using the pROC package in R.

### 2.4 Nomogram performance

The performance of the predictive nomogram was assessed based on discriminatory power, calibration, and clinical utility. The ability of a model to correctly distinguish between events and non-events is called discrimination. to We evaluated the recognition efficiency of the predictive nomograms using ROC curves (21). Calibration measures the extent to which the predicted probabilities matched the actual results. We assessed calibration capability using Hosmer–Lemeshow tests with p > 0.05 indicating satisfactory calibration (22). A nomogram map was created to further evaluate the calibration. Internal verification proceeded by bootstrapping samples1,000 times (23). The clinical effectiveness of the predictive nomograms was evaluated based on the net benefit of different threshold probabilities using decision curve analysis (24). The optimal cutoff value was determined when the Youden index (sensitivity + specificity-1was maximal based on the results of ROC curve analyses of the training cohort.

## 3 Results

### 3.1 Characteristics of the patients

**Figure 1:**
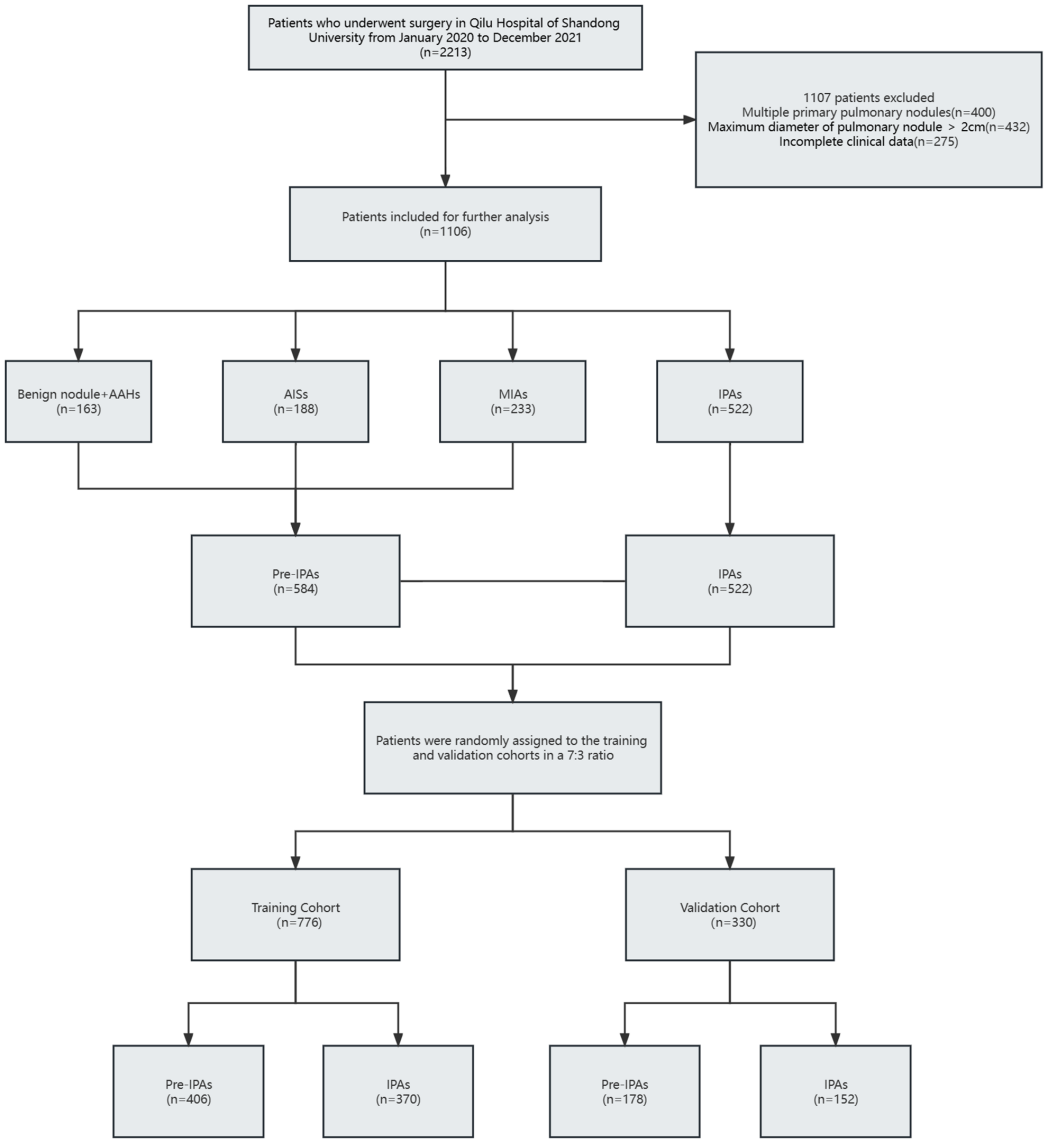
Flow diagram of patient selection process. AAH, atypical adenomatous hyperplasia; AIS, adenocarcinoma *in situ*; IPA, invasive pulmonary adenocarcinoma.; MIA, microinvasive adenocarcinoma.

2213 original patients who had surgery at our institution between January 2020 and December 2021 were included in our research. The initial patients were not chosen; they were all sequential. Following a series of screening steps, 1,106 suitable patients were eventually enrolled in our research. **Figure 1** shows the process of identifying and selecting 1,106 eligible patients, among whom 163, 188, 233, and 522 had benign nodules and AAH, AIS, MIA, and IPA, respectively. All patients were assigned to pre-IPA (n = 584) and IPA (n = 522) groups based on nodule invasiveness. The patients were then randomly assigned to training (n = 776) or validation (n = 330) cohort at a 7:3 ratio. with No variables significantly differed between the cohorts (**Table 1**). The training cohort comprised 406 and 370 patients with pre-IPA and IPA nodules and the validation cohort comprised 178 and 152 patients with pre-IPA and IPA nodules, respectively. **Table 2** shows he characteristics of the patients in the training and validation groups.

**Table 1:** Characteristics of patients in training and validation cohorts.

Characteristics	All cohort (N=1106)	Validation cohort (N=330)	Training cohort (N=776)	P
IPA, n (%)				0.621
No	584 (52.8)	178 (53.9)	406 (52.3)	
Yes	522 (47.2)	152 (46.1)	370 (47.7)	
Gender, n (%)				0.134
Female	663 (59.9)	209 (63.3)	454 (58.5)	
Male	443 (40.1)	121 (36.7)	322 (41.5)	
Hypertension, n (%)				0.147
No	798 (72.2)	248 (75.2)	550 (70.9)	
Yes	308 (27.8)	82 (24.8)	226 (29.1)	
Diabetes, n (%)				0.752
No	970 (87.7)	291 (88.2)	679 (87.5)	
Yes	136 (12.3)	39 (11.8)	97 (12.5)	
COPD, n (%)				0.162
No	1096 (99.1)	325 (98.5)	771 (99.4)	
Yes	10 (0.9)	5 (1.5)	5 (0.6)	
Smoking history, n (%)				0.069
Non-smoker	856 (77.4)	267 (80.9)	589 (75.9)	
Smoker	250 (22.6)	63 (19.1)	187 (24.1)	
Blood type, n (%)				0.863
A	336 (30.4)	106 (32.1)	230 (29.6)	
B	373 (33.7)	110 (33.3)	263 (33.9)	
AB	127 (11.5)	37 (11.2)	90 (11.6)	
O	270 (24.4)	77 (23.3)	193 (24.9)	
ASA, n (%)				0.775
1	123 (11.1)	40 (12.1)	83 (10.7)	
2	954 (86.3)	281 (85.2)	673 (86.7)	
3	29 (2.6)	9 (2.7)	20 (2.6)	
Location, n (%)				0.217
Central	102 (9.2)	25 (7.6)	77 (9.9)	
Peripheral	1004 (90.8)	305 (92.4)	699 (90.1)	
Shape, n (%)				0.77
Regular	537 (48.6)	158 (47.9)	379 (48.8)	
Irregular	569 (51.4)	172 (52.1)	397 (51.2)	
Spiculation, n (%)				0.272
No	495 (44.8)	156 (47.3)	339 (43.7)	
Yes	611 (55.2)	174 (52.7)	437 (56.3)	
Cavitation sign, n (%)				0.616
No	931 (84.2)	275 (83.3)	656 (84.5)	
Yes	175 (15.8)	55 (16.7)	120 (15.5)	
Calcification, n (%)				0.629
No	1092 (98.7)	325 (98.5)	767 (98.8)	
Yes	14 (1.3)	5 (1.5)	9 (1.2)	
Vascular penetration sign, n (%)				0.734
No	387 (35.0)	113 (34.2)	274 (35.3)	
Yes	719 (65.0)	217 (65.8)	502 (64.7)	
Pleural adhesions, n (%)				0.731
No	561 (50.7)	170 (51.5)	391 (50.4)	
Yes	545 (49.3)	160 (48.5)	385 (49.6)	
Bronchus sign, n (%)				0.751
No	868 (78.5)	257 (77.9)	611 (78.7)	
Yes	238 (21.5)	73 (22.1)	165 (21.3)	
Lobulation, n (%)				0.996
No	714 (64.6)	213 (64.5)	501 (64.6)	
Yes	392 (35.4)	117 (35.5)	275 (35.4)	
Lymph node enlargement sign, n (%)				0.197
No	949 (85.8)	290 (87.9)	659 (84.9)	
Yes	157 (14.2)	40 (12.1)	117 (15.1)	
Pleural effusion sign, n (%)				0.764
No	1098 (99.3)	328 (99.4)	770 (99.2)	
Yes	8 (0.7)	2 (0.6)	6 (0.8)	
Albumin (g/L), median (IQR)	59.90 (57.70–62.10)	59.75 (57.52–61.90)	60.00 (57.80–62.10)	0.308
Lymphocyte (×109/L), median (IQR)	1.80 (1.47–2.21)	1.73 (1.43–2.19)	1.84 (1.50–2.21)	0.058
PNI (%), median (IQR)	69.18 (66.25–71.85)	68.85 (65.85–71.11)	69.28 (66.44–72.11)	0.061
Neutrophil (×109/L), median (IQR)	2.99 (2.45–3.75)	2.91 (2.36–3.76)	3.01 (2.49–3.75)	0.206
Eosinophil (×109/L), median (IQR)	0.10 (0.06–0.17)	0.10 (0.07–0.17)	0.10 (0.06–0.17)	0.639
Basophil (×109/L), median (IQR)	0.03 (0.02–0.04)	0.03 (0.02–0.04)	0.03 (0.02–0.04)	0.622
Monocyte (×109/L), median (IQR)	0.41 (0.34–0.50)	0.41 (0.33–0.50)	0.41 (0.34–0.50)	0.97
Erythrocyte (×1012/L), median (IQR)	4.49 (4.19–4.82)	4.49 (4.16–4.77)	4.49 (4.20–4.83)	0.59
Hemoglobin (g/L), median (IQR)	137.00 (128.00–148.00)	135.00 (128.00–145.75)	137.00 (128.00–148.00)	0.316
Platelet (×109/L), median (IQR)	235.00 (200.00–270.00)	234.00 (197.25–264.75)	236.00 (201.00–271.00)	0.64
NLR (%), median (IQR)	1.67 (1.29–2.12)	1.65 (1.28–2.16)	1.68 (1.30–2.12)	0.93
PLR (%), median (IQR)	130.18 (104.77–158.70)	134.83 (103.57, 164.60)	128.57 (104.95, 156.87)	0.087
MLR (%), median (IQR)	0.22 (0.18–0.28)	0.23 (0.19–0.29)	0.22 (0.18–0.28)	0.152
dNLR (%), median (IQR)	1.26 (1.00–1.56)	1.25 (1.00–1.57)	1.26 (1.01–1.56)	0.581
NLPR (%), median (IQR)	0.01 (0.01–0.01)	0.01 (0.01–0.01)	0.01 (0.01–0.01)	0.872
SIRI (%), median (IQR)	0.66 (0.48–0.96)	0.66 (0.47–0.99)	0.66 (0.48–0.95)	0.867
AISI (%), median (IQR)	153.06 (104.71–232.70)	150.72 (99.01–251.10)	154.28 (105.92–226.32)	0.722
SII (%), median (IQR)	382.56 (289.63–515.89)	385.65 (280.19–533.38)	382.43 (293.91–506.56)	0.828
PIV (%), median (IQR)	153.06 (104.71–232.70)	150.72 (99.01–251.10)	154.28 (105.92–226.32)	0.722
Blood sugar(mmol/L), median (IQR)	5.12 (4.73–5.66)	5.14 (4.75–5.61)	5.11 (4.72–5.67)	0.949
Complement C1q(mg/L), median (IQR)	171.60 (151.67, 191.28)	172.05 (150.52, 190.82)	171.50 (152.60, 191.33)	0.654
LDH (U/L), median (IQR)	192.00 (172.00–215.00)	193.00 (174.00–215.75)	191.00 (171.00–215.00)	0.192
SA (mg/dL), median (IQR)	53.90 (49.30–58.20)	53.10 (49.30–57.75)	54.03 (49.38–58.30)	0.266
5’-NT (U/L), median (IQR)	4.00 (3.00–5.00)	4.00 (3.00–5.00)	4.00 (3.00–5.00)	0.62
Pro-GRP (pg/mL), median (IQR)	41.96 (34.08–45.92)	41.96 (34.46–46.25)	41.96 (33.72–45.55)	0.941
SCC (ng/mL), median (IQR)	1.08 (0.80–1.97)	1.07 (0.80–1.70)	1.08 (0.80–1.97)	0.666
Cyfra21–1 (ng/mL), median (IQR)	2.32 (1.69–2.56)	2.31 (1.68–2.57)	2.32 (1.70–2.56)	0.688
CEA (ng/mL), median (IQR)	2.32 (1.51–2.64)	2.29 (1.46, 2.74)	2.32 (1.53, 2.62)	0.303
CA125 (U/mL), median (IQR)	10.72 (7.61–11.38)	10.71 (7.59–11.90)	10.72 (7.62–11.20)	0.703
NSE (ng/mL), median (IQR)	19.45 (15.80–20.50)	19.10 (15.30–20.38)	19.45 (16.20–20.62)	0.149
Age (years), median (IQR)	57.00 (50.00–65.00)	58.00 (51.25–64.75)	57.00 (50.00–65.00)	0.472
BMI (kg/m2), median (IQR)	24.77 (22.77–26.90)	24.65 (22.58–26.88)	24.83 (22.90–26.93)	0.209
FEV1% predicted (%), median (IQR)	105.32 (94.89–115.71)	105.29 (93.37–116.07)	105.32 (95.03–115.59)	0.98
MVV% predicted (%), median (IQR)	104.36 (90.42–116.43)	105.16 (91.07–116.69)	104.06 (90.29–116.35)	0.248
Maximum diameter (cm), median (IQR)	1.20 (0.80–1.50)	1.10 (0.80–1.50)	1.20 (0.80–1.50)	0.075
CTR (%), median (IQR)	0.00 (0.00–0.71)	0.00 (0.00–0.65)	0.00 (0.00–0.74)	0.172

IPA, invasive pulmonary adenocarcinoma; COPD, chronic obstructive pulmonary diseases; ASA, American Society of Anesthesiologists; PNI, prognostic nutritional index; NLR, neutrophil-lymphocyte ratio; PLR, platelet-lymphocyte ratio; MLR, monocyte-lymphocyte ratio; dNLR, derived neutrophil-to-lymphocyte ratio; NLPR, neutrophil to lymphocyte and platelet ratio; SIRI, systemic inflammatory response syndrome; AISI, aggregate index of systemic inflammation; SII, systemic inflammation index; PIV, pan-immune-inflammation value; LDH, lactate dehydrogenase; SA, serum amyloid; 5’-NT, 5’-nucleotidase; Pro-GRP, pro-gastrin-releasing peptide; SCC, squamous cell carcinoma; Cyfra21–1, cytokeratin 19-fragments; CEA, carcinoembryonic antigen; CA125, carcinoma antigen 125; NSE, neuron-specific enolase; BMI, body mass index; FEV1, forced expiratory volume in one second; MVV, maximal voluntary ventilation; CTR, consolidation-to-tumor ratio.

P-value for the comparison between the training and validation cohorts.

**Table 2:** Clinical characteristics of patients with IPA and pre-IPA in the training and validation cohorts.

Characteristics	Training cohort(n=776)			Validation cohort(n=330)		
	Pre-IPA (n=406)	IPA (n=370)	p	Pre-IPA (n=178)	IPA (n=152)	P value
Gender, n (%)			0.003			0.058
Female	258 (63.5)	196 (53.0)		121 (68.0)	88 (57.9)	
Male	148 (36.5)	174 (47.0)		57 (32.0)	64 (42.1)	
Hypertension, n (%)			0.024			0.009
No	302 (74.4)	248 (67.0)		144 (80.9)	104 (68.4)	
Yes	104 (25.6)	122 (33.0)		34 (19.1)	48 (31.6)	
Diabetes, n (%)			0.55			0.723
No	358 (88.2)	321 (86.8)		158 (88.8)	133 (87.5)	
Yes	48 (11.8)	49 (13.2)		20 (11.2)	19 (12.5)	
COPD, n (%)			0.58			0.529
No	404 (99.5)	367 (99.2)		176 (98.9)	149 (98.0)	
Yes	2 (0.5)	3 (0.8)		2 (1.1)	3 (2.0)	
Smoking history, n (%)			<0.001			0.005
Non-smoker	329 (81.0)	260 (70.3)		154 (86.5)	113 (74.3)	
Smoker	77 (19.0)	110 (29.7)		24 (13.5)	39 (25.7)	
Blood type, n (%)			0.254			0.626
A	131 (32.3)	99 (26.8)		55 (30.9)	51 (33.6)	
B	126 (31.0)	137 (37.0)		56 (31.5)	54 (35.5)	
AB	48 (11.8)	42 (11.4)		21 (11.8)	16 (10.5)	
O	101 (24.9)	92 (24.9)		46 (25.8)	31 (20.4)	
ASA, n (%)			0.008			0.019
1	54 (13.3)	29 (7.8)		28 (15.7)	12 (7.9)	
2	346 (85.2)	327 (88.4)		148 (83.1)	133 (87.5)	
3	6 (1.5)	14 (3.8)		2 (1.1)	7 (4.6)	
Location, n (%)			0.046			0.061
Central	32 (7.9)	45 (12.2)		9 (5.1)	16 (10.5)	
Peripheral	374 (92.1)	325 (87.8)		169 (94.9)	136 (89.5)	
Shape, n (%)			<0.001			<0.001
Regular	253 (62.3)	126 (34.1)		109 (61.2)	49 (32.2)	
Irregular	153 (37.7)	244 (65.9)		69 (38.8)	103 (67.8)	
Spiculation, n (%)			<0.001			<0.001
No	229 (56.4)	110 (29.7)		104 (58.4)	52 (34.2)	
Yes	177 (43.6)	260 (70.3)		74 (41.6)	100 (65.8)	
Cavitation sign, n (%)			<0.001			0.004
No	361 (88.9)	295 (79.7)		158 (88.8)	117 (77.0)	
Yes	45 (11.1)	75 (20.3)		20 (11.2)	35 (23.0)	
Calcification, n (%)			0.124			0.529
No	399 (98.3)	368 (99.5)		176 (98.9)	149 (98.0)	
Yes	7 (1.7)	2 (0.5)		2 (1.1)	3 (2.0)	
Vascular penetration sign, n (%)			<0.001			0.005
No	174 (42.9)	100 (27.0)		73 (41.0)	40 (26.3)	
Yes	232 (57.1)	270 (73.0)		105 (59.0)	112 (73.7)	
Pleural adhesions, n (%)			<0.001			<0.001
No	261 (64.3)	130 (35.1)		110 (61.8)	60 (39.5)	
Yes	145 (35.7)	240 (64.9)		68 (38.2)	92 (60.5)	
Bronchus sign, n (%)			<0.001			<0.001
No	361 (88.9)	250 (67.6)		155 (87.1)	102 (67.1)	
Yes	45 (11.1)	120 (32.4)		23 (12.9)	50 (32.9)	
Lobulation, n (%)			<0.001			<0.001
No	318 (78.3)	183 (49.5)		134 (75.3)	79 (52.0)	
Yes	88 (21.7)	187 (50.5)		44 (24.7)	73 (48.0)	
Lymph node enlargement sign, n (%)			<0.001			0.226
No	363 (89.4)	296 (80.0)		160 (89.9)	130 (85.5)	
Yes	43 (10.6)	74 (20.0)		18 (10.1)	22 (14.5)	
Pleural effusion sign, n (%)			0.35			0.125
No	404 (99.5)	366 (98.9)		178 (100.0)	150 (98.7)	
Yes	2 (0.5)	4 (1.1)		0 (0.0)	2 (1.3)	
Albumin (g/L), median (IQR)	59.85 (57.50–62.10)	60.10 (58.10–62.20)	0.275	59.75 (57.73–61.60)	59.80 (57.40–62.20)	0.809
Lymphocyte (×109/L), median (IQR)	1.85 (1.53–2.24)	1.79 (1.46–2.21)	0.183	1.75 (1.46–2.23)	1.71 (1.37–2.14)	0.168
PNI (%), median (IQR)	69.30 (66.55–72.15)	69.22 (66.25–72.05)	0.846	68.90 (66.45–71.20)	68.80 (65.59–70.96)	0.386
Neutrophil (×109/L), median (IQR)	3.00 (2.45–3.61)	3.01 (2.49–3.89)	0.457	2.87 (2.30–3.64)	2.95 (2.43–3.86)	0.239
Eosinophil (×109/L), median (IQR)	0.10 (0.06–0.16)	0.11 (0.06–0.18)	0.151	0.10 (0.06–0.16)	0.11 (0.07–0.20)	0.129
Basophil (×109/L), median (IQR)	0.03 (0.02–0.04)	0.03 (0.02–0.04)	0.135	0.03 (0.02–0.04)	0.03 (0.02–0.04)	0.645
Monocyte (×109/L), median (IQR)	0.40 (0.34–0.49)	0.42 (0.34–0.51)	0.301	0.39 (0.33–0.49)	0.42 (0.35–0.50)	0.196
Erythrocyte (×1012/L), median (IQR)	4.47 (4.18–4.81)	4.50 (4.22–4.84)	0.516	4.46 (4.16–4.74)	4.50 (4.16–4.81)	0.461
Hemoglobin (g/L), median (IQR)	136.00 (128.00–147.00)	138.50 (128.00–149.00)	0.298	134.00 (128.00–144.00)	137.00 (128.00–148.00)	0.199
Platelet (×109/L), median (IQR)	236.00 (205.00–273.00)	235.50 (198.25–269.00)	0.599	236.50 (196.50–270.75)	232.50 (198.50–258.25)	0.583
NLR (%), median (IQR)	1.63 (1.31–2.08)	1.73 (1.29–2.19)	0.149	1.61 (1.26–1.99)	1.71 (1.32–2.38)	0.074
PLR (%), median (IQR)	125.91 (106.66–152.62)	131.16 (103.89–160.19)	0.329	134.32 (102.34–158.85)	135.09 (104.23–173.10)	0.335
MLR (%), median (IQR)	0.22 (0.18–0.27)	0.23 (0.18–0.29)	0.06	0.22 (0.18–0.28)	0.24 (0.20–0.31)	0.019
dNLR (%), median (IQR)	1.24 (1.01–1.54)	1.30 (1.01–1.58)	0.332	1.22 (0.99–1.48)	1.27 (1.01–1.63)	0.247
NLPR (%), median (IQR)	0.01 (0.01–0.01)	0.01 (0.01–0.01)	0.095	0.01 (0.01–0.01)	0.01 (0.01–0.01)	0.039
SIRI (%), median (IQR)	0.64 (0.48, 0.92)	0.68 (0.49, 0.97)	0.156	0.63 (0.46, 0.90)	0.74 (0.48, 1.12)	0.018
AISI (%), median (IQR)	148.80 (105.11–212.73)	160.39 (106.61–237.09)	0.143	139.25 (94.96–247.98)	170.02 (109.58–253.37)	0.075
SII (%), median (IQR)	376.33 (289.58–487.56)	392.56 (296.72–524.88)	0.16	379.73 (269.92–518.01)	410.20 (295.55–551.70)	0.232
PIV (%), median (IQR)	148.80 (105.11–212.73)	160.39 (106.61–237.09)	0.143	139.25 (94.96–247.98)	170.02 (109.58–253.37)	0.075
Blood sugar(mmol/L), median (IQR)	5.04 (4.70–5.58)	5.20 (4.75–5.80)	0.016	5.10 (4.75–5.51)	5.18 (4.75–5.85)	0.174
Complement C1q(mg/L), median (IQR)	171.40 (154.10–191.00)	172.05 (150.60–191.70)	0.87	169.45 (150.43–190.67)	174.15 (151.38–190.20)	0.408
LDH (U/L), median (IQR)	190.00 (171.00–214.00)	192.00 (171.00–216.00)	0.538	191.00 (171.25–209.50)	195.94 (177.75–222.00)	0.037
SA (mg/dL), median (IQR)	53.80 (49.12–57.27)	54.06 (49.95–59.18)	0.038	52.45 (48.95–56.70)	54.03 (49.58–58.70)	0.044
5’-NT (U/L), median (IQR)	4.00 (3.00–5.00)	4.00 (3.00–5.00)	0.65	4.00 (3.00–5.00)	4.00 (3.00–5.00)	0.691
Pro-GRP (pg/mL), median (IQR)	41.96 (33.02, 45.11)	41.96 (35.34–46.06)	0.088	41.96 (34.75–47.35)	41.83 (33.63–45.55)	0.383
SCC (ng/mL), median (IQR)	1.06 (0.80–1.94)	1.10 (0.77–1.97)	0.664	1.01 (0.74–1.70)	1.13 (0.80–1.72)	0.292
Cyfra21–1 (ng/mL), median (IQR)	2.32 (1.62–2.53)	2.32 (1.79–2.58)	0.102	2.18 (1.61–2.39)	2.32 (1.85–2.65)	0.05
CEA (ng/mL), median (IQR)	2.28 (1.35–2.45)	2.32 (1.84–2.96)	<0.001	2.17 (1.31–2.42)	2.32 (1.61–3.03)	0.051
CA125 (U/mL), median (IQR)	10.72 (7.61–11.30)	10.72 (7.62–11.07)	0.936	10.72 (7.56–11.85)	10.05 (7.68–11.98)	0.627
NSE (ng/mL), median (IQR)	19.45 (16.42–20.50)	19.45 (15.80–20.70)	0.62	19.15 (15.20–20.48)	19.05 (15.50–20.02)	0.837
Age (years), median (IQR)	56.00 (47.00–62.00)	60.00 (54.00–67.00)	<0.001	56.00 (48.00–61.00)	62.00 (54.75–68.00)	<0.001
BMI (kg/m2), median (IQR)	24.55 (22.61–26.44)	25.18 (23.15–27.23)	0.005	24.59 (22.24–26.49)	24.82 (22.86–27.00)	0.101
FEV1% predicted (%), median (IQR)	105.98 (96.53, 115.24)	104.32 (93.81, 115.71)	0.212	105.61 (96.52–114.50)	104.51 (91.76–117.44)	0.791
MVV% predicted (%), median (IQR)	104.27 (91.98–116.54)	103.94 (88.44–115.34)	0.444	106.61 (94.15–120.17)	104.38 (88.08–115.12)	0.181
Maximum diameter (cm), median (IQR)	1.00 (0.70–1.30)	1.50 (1.20–1.80)	<0.001	1.00 (0.70–1.20)	1.50 (1.20–1.70)	<0.001
CTR (%), median (IQR)	0.00 (0.00–0.38)	0.54 (0.00–0.92)	<0.001	0.00 (0.00–0.43)	0.41 (0.00–0.78)	<0.001

IPA, invasive pulmonary adenocarcinoma; COPD, chronic obstructive pulmonary diseases; ASA, American Society of Anesthesiologists; PNI, prognostic nutritional index; NLR, neutrophil-lymphocyte ratio; PLR, platelet-lymphocyte ratio; MLR, monocyte-lymphocyte ratio; dNLR, derived neutrophil-to-lymphocyte ratio; NLPR, neutrophil-to-lymphocyte and platelet ratio; SIRI, systemic inflammatory response syndrome; AISI, aggregate index of systemic inflammation; SII, systemic inflammation index; PIV, pan-immune-inflammation value; LDH, lactate dehydrogenase; SA, serum amyloid; 5’-NT, 5’-nucleotidase; Pro-GRP, pro-gastrin-releasing peptide; SCC, squamous cell carcinoma; Cyfra21–1, cytokeratin 19-fragments; CEA, carcinoembryonic antigen; CA125, carcinoma antigen 125; NSE, neuron-specific enolase; BMI, body mass index; FEV1, forced expiratory volume in one second; MVV, maximal voluntary ventilation; CTR, consolidation-to-tumor ratio.

### 3.2 Identification of risk factors for SPN aggressiveness measuring ≤ 2 cm

**Figure 2:**
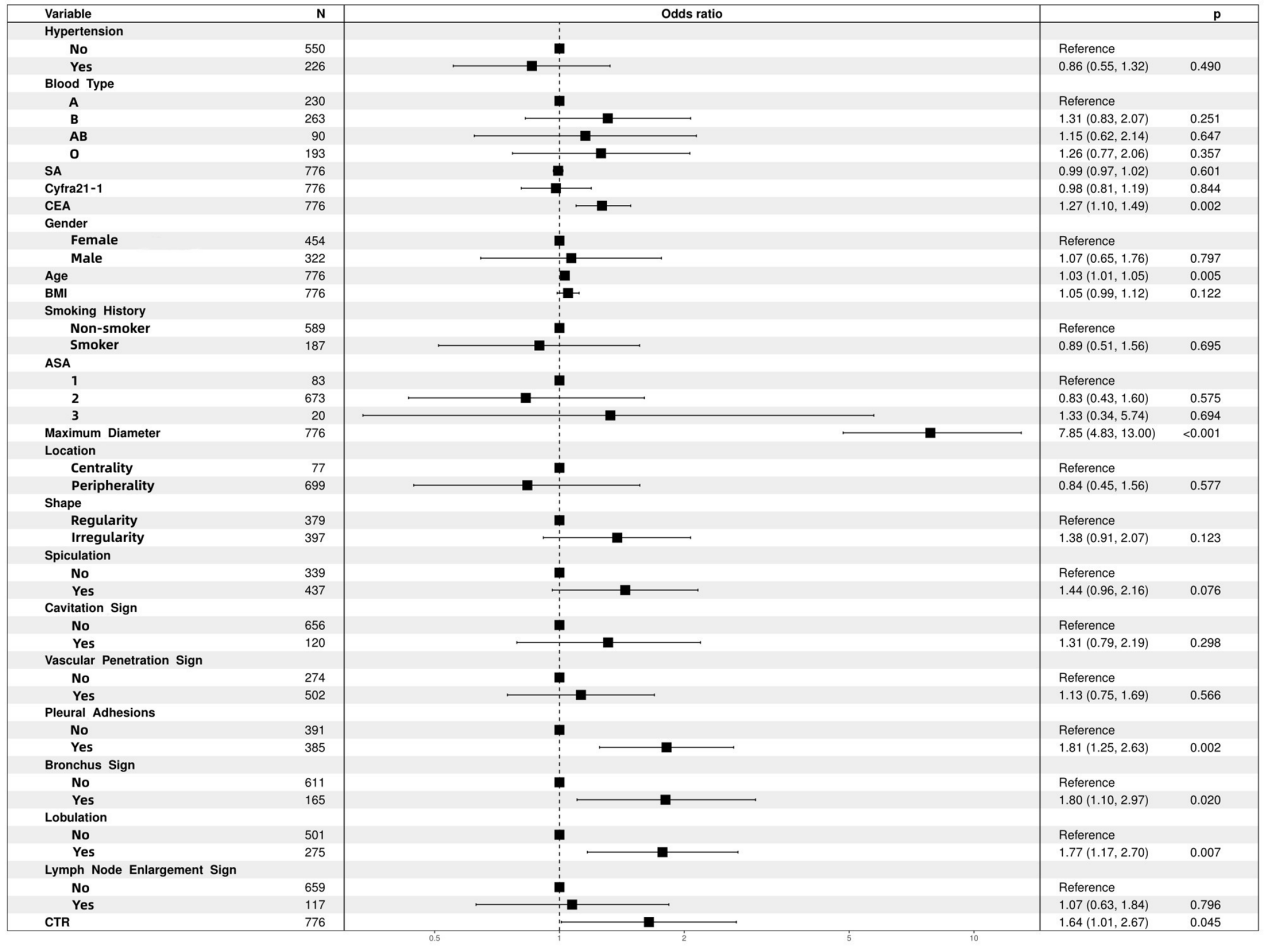
Multifactor logistic regression analysis of forest plots. ASA, American Society of Anesthesiologists; BMI, body mass index; CEA, carcinoembryonic antigen; CTR, consolidation-to-tumor ratio; SA, serum amyloid.

We identified independent risk factors for IPA in the training cohort using univariate and multivariate logistic regression analyses (**Table 3**) Univariate analysis revealed 21 potential risk factors for IPA within 2 cm (p < 0.05). These 21 factors (p < 0.05) were further analyzed using multivariate logistic regression, and the following indicators were selected: age (odds ratio [OR)], 1.030; 95% confidence interval [CI], 1.009–1.052; p = 0.005); CEA (OR,1.267; 95% CI, 1.097–1.486; p = 0.002); bronchus involvement (yes *vs*. no; OR, 1.802; 95% CI, 1.103–2.972; p = 0.02); lobulation (yes *vs*. no; OR, 1.772; 95% CI, 1.167–2.696; p = 0.007); pleural adhesions (yes *vs*. no; OR, 1.813; 95% CI, 1.250–2.632; p = 0.002); maximum tumor diameter (OR, 7.848; 95% CI: 4.834–13.003; p < 0.001); and CTR (OR, 1.644; 95% CI, 1.011–2.671; p = 0.045; **Figure 2**, forest plot).

**Table 3:** Univariate and multivariate logistic regression analysis of IPA factors of SPN ≤2 cm in the training cohort.

Variables	Univariate analysis		Multivariate analysis	
	OR (95% CI)	P value	OR (95% CI)	P value
Age	1.052 (1.037–1.068)	<0.001	1.030 (1.009–1.052)	0.005
Bronchus sign				
No	Ref.	Ref.	Ref.	Ref.
Yes	3.851 (2.655–5.669)	<0.001	1.802 (1.103–2.972)	0.02
Cavitation sign				
No	Ref.	Ref.	Ref.	Ref.
Yes	2.040 (1.372–3.061)	<0.001	1.310 (0.789–2.189)	0.298
CEA	1.410 (1.248–1.609)	<0.001	1.267 (1.097–1.486)	0.002
CTR	5.184 (3.584–7.568)	<0.001	1.644 (1.011–2.671)	0.045
Lobulation				
No	Ref.	Ref.	Ref.	Ref.
Yes	3.693 (2.711–5.061)	<0.001	1.772 (1.167–2.696)	0.007
Lymph node enlargement sign				
No	Ref.	Ref.	Ref.	Ref.
Yes	2.110 (1.413–3.188)	<0.001	1.073 (0.629–1.836)	0.796
Maximum diameter	16.887 (11.033–26.474)	<0.001	7.848 (4.834–13.003)	<0.001
Pleural adhesions				
No	Ref.	Ref.	Ref.	Ref.
Yes	3.323 (2.480–4.471)	<0.001	1.813 (1.250–2.632)	0.002
Shape				
Regular	Ref.	Ref.	Ref.	Ref.
Irregular	3.202 (2.391–4.307)	<0.001	1.378 (0.915–2.072)	0.123
Spiculation				
No	Ref.	Ref.	Ref.	Ref.
Yes	3.058 (2.277–4.125)	<0.001	1.441 (0.961–2.158)	0.076
Vascular penetration sign				
No	Ref.	Ref.	Ref.	Ref.
Yes	2.025 (1.500–2.745)	<0.001	1.127 (0.749–1.695)	0.566
Smoking history				
Non-smoker	Ref.	Ref.	Ref.	Ref.
Smoker	1.808 (1.297–2.529)	0.001	0.895 (0.511–1.561)	0.695
BMI	1.070 (1.023–1.119)	0.003	1.049 (0.987–1.115)	0.122
Gender				
Female	Ref.	Ref.	Ref.	Ref.
Male	1.548 (1.162–2.064)	0.003	1.068 (0.646–1.762)	0.797
ASA				
1	Ref.	Ref.	Ref.	Ref.
2	1.760 (1.102–2.864)	0.02	0.829 (0.433–1.603)	0.575
3	4.345 (1.568–13.399)	0.006	1.327 (0.336–5.736)	0.694
Hypertension				
No	Ref.	Ref.	Ref.	Ref.
Yes	1.429 (1.047–1.951)	0.025	0.858 (0.554–1.324)	0.49
Cyfra21_1	1.188 (1.023–1.389)	0.026	0.981 (0.809–1.193)	0.844
SA	1.022 (1.001–1.043)	0.041	0.993 (0.966–1.020)	0.601
Blood type				
A	Ref.	Ref.	Ref.	Ref.
B	1.439 (1.009–2.056)	0.045	1.308 (0.827–2.073)	0.251
AB	1.158 (0.708–1.889)	0.557	1.155 (0.623–2.140)	0.647
O	1.205 (0.821–1.772)	0.341	1.260 (0.771–2.064)	0.357
Location				
Central	Ref.	Ref.	Ref.	Ref.
Peripheral	0.618 (0.381–0.992)	0.048	0.837 (0.445–1.563)	0.577
Blood sugar	1.101 (0.979–1.244)	0.112		
FEV1 predicted	0.993 (0.985–1.002)	0.112		
NLR	1.133 (0.974–1.349)	0.132		
Calcification				
No	Ref.	Ref.		
Yes	0.310 (0.046–1.291)	0.145		
SIRI	1.100 (0.979–1.284)	0.149		
Pro-GRP	1.007 (0.997–1.018)	0.172		
Monocyte	1.248 (0.955, 1.903)	0.174		
AISI	1.000 (1.000–1.001)	0.179		
PIV	1.000 (1.000–1.001)	0.179		
SII	1.000 (1.000–1.001)	0.226		
PLR	1.002 (0.999–1.005)	0.258		
dNLR	1.144 (0.897–1.480)	0.287		
Eosinophil	1.591 (0.698–4.269)	0.301		
MLR	1.259 (0.841–2.208)	0.312		
Pleural effusion sign				
No	Ref.	Ref.		
Yes	2.208 (0.428–15.993)	0.362		
SCC	1.093 (0.903–1.336)	0.367		
IDH	1.002 (0.998–1.005)	0.376		
Basophil	4.820 (0.222–505.195)	0.377		
Neutrophil	1.041 (0.931–1.168)	0.477		
Diabetes				
No	Ref.	Ref.		
Yes	1.138 (0.743–1.745)	0.55		
Lymphocyte	0.935 (0.736–1.187)	0.583		
COPD				
No	Ref.	Ref.		
Yes	1.651 (0.272–12.588)	0.584		
Hemoglobin	1.003 (0.993–1.012)	0.59		
Platelet	0.999 (0.997–1.002)	0.593		
MVV predicted	0.999 (0.992–1.005)	0.654		
PNI	0.995 (0.968–1.022)	0.723		
CA125	1.003 (0.978–1.028)	0.828		
Erythrocyte	1.030 (0.761–1.394)	0.848		
Complement_c1q	1.000 (0.996–1.004)	0.949		
Albumin	0.999 (0.969–1.031)	0.962		
5’-NT	1.002 (0.917–1.094)	0.963		
NSE	1.000 (0.977–1.023)	0.992		

IPA, invasive pulmonary adenocarcinoma; COPD, chronic obstructive pulmonary diseases; ASA, American Society of Anesthesiologists; PNI, prognostic nutritional index; NLR, neutrophil-lymphocyte ratio; PLR, platelet-lymphocyte ratio; MLR, monocyte-lymphocyte ratio; dNLR, derived neutrophil-to-lymphocyte ratio; NLPR, neutrophil to lymphocyte and platelet ratio; SIRI, systemic inflammatory response syndrome; AISI, aggregate index of systemic inflammation; SII, systemic inflammation index; PIV, pan-immune-inflammation value; LDH, lactate dehydrogenase; SA, serum amyloid; 5’-NT, 5’-nucleotidase; Pro-GRP, pro-gastrin-releasing peptide; SCC, squamous cell carcinoma; Cyfra21–1, cytokeratin 19-fragments; CEA, carcinoembryonic antigen; CA125, carcinoma antigen 125; NSE, neuron-specific enolase; BMI, body mass index; FEV1, forced expiratory volume in one second; MVV, maximal voluntary ventilation; CTR, consolidation-to-tumor ratio; OR, odds ratio; CI, confidence interval.

### 3.3 Nomogram construction

The seven independent risk factors were modeled using logistic regression. **Table 4** shows details of the predictive models. Thus, invasiveness of SPN ≤ 2 cm was predicted as follows:

**Table 4:** Details of the predictive model used to calculate the probability of IPA for SPN measuring ≤2 cm in diameter.

Risk factors	Estimate	Std. Error	Statistic	OR (95% CI)	P value
Intercept	-3.766	1.532		0.023	0.014
Maximum diameter	2.06	0.252	8.174	7.848 (4.834–13.003)	<0.001
CTR	0.497	0.248	2.008	1.644 (1.011–2.671)	0.045
Age	0.03	0.011	2.801	1.030 (1.009–1.052)	0.005
CEA	0.236	0.078	3.046	1.267 (1.097–1.486)	0.002
Pleural adhesions					
No	Ref.				
Yes	0.595	0.19	3.135	1.813 (1.250–2.632)	0.002
Lobulation					
No	Ref.				
Yes	0.572	0.213	2.684	1.772 (1.167–2.696)	0.007
Bronchus sign					
No	Ref.				
Yes	0.589	0.252	2.333	1.802 (1.103–2.972)	0.02

IPA, invasive pulmonary adenocarcinoma; CEA, carcinoembryonic antigen; CTR, consolidation-to-tumor ratio; OR, odds ratio; CI, confidence interval.

Predicted IPA = e^x^/(1 + e^x^), x = 2.06 × maximum diameter + 0.497 × CTR + 0.03×age + 0.236 × CEA + 0.595 × pleural adhesions (no = 0; yes = 1) + 0.572 × lobulation (no = 0; yes = 1) + 0.589 × bronchus sign (no = 0; yes = 1)-3.766.

Where e is the natural logarithmic base, e = 2.718 281 828, and x is the logistic regression coefficient. The units of maximum diameter, age, and CEA are cm, years, and ng/mL, respectively.

**Figure 3:**
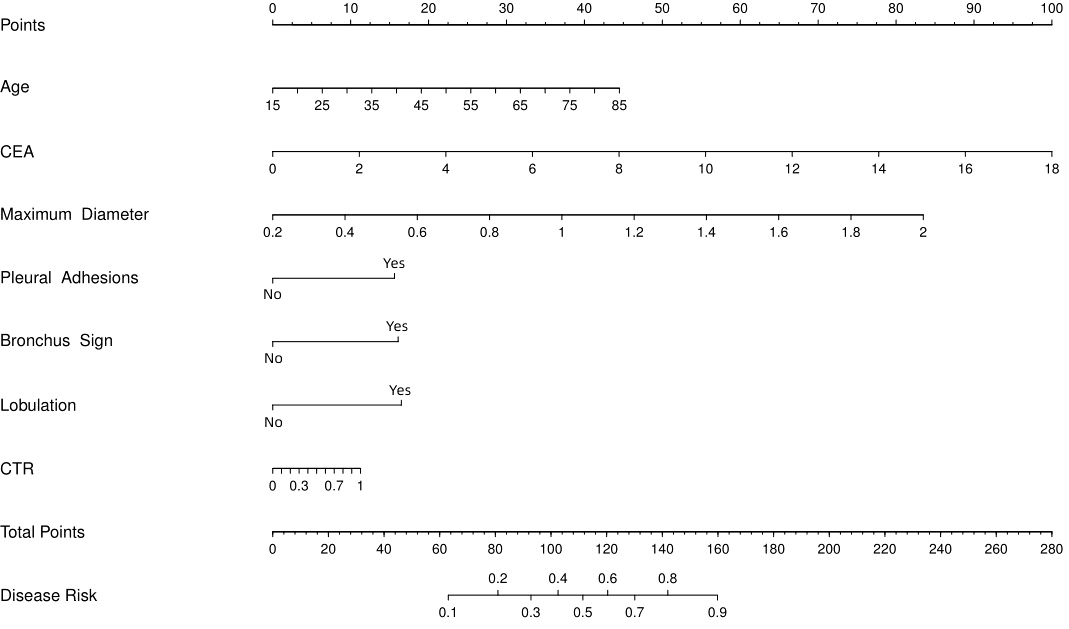
Nomogram to predict probability of IPA for SPN ≤ 2 cm. CEA, carcinoembryonic antigen; CTR, consolidation-to-tumor ratio.

Based on the coefficients of the multiple logistic regression model, a nomogram predicting the IPA of SPN ≤ 2 cm was drawn using the rms package in R (**Figure 3**). This nomogram comprised 10 axes, of which axes 2–8 represent the seven variables in the prediction model. By plotting a line perpendicular to the highest point axis, the estimated score for each risk factor was calculated and summed to obtain the total score. The total point axis was used to predict the probability of preoperative IPA of SPNs measuring ≤ 2 cm. An appropriate surgical method could then be selected.

### 3.4 Predictive performance and validation of the nomogram

**Figure 4:**
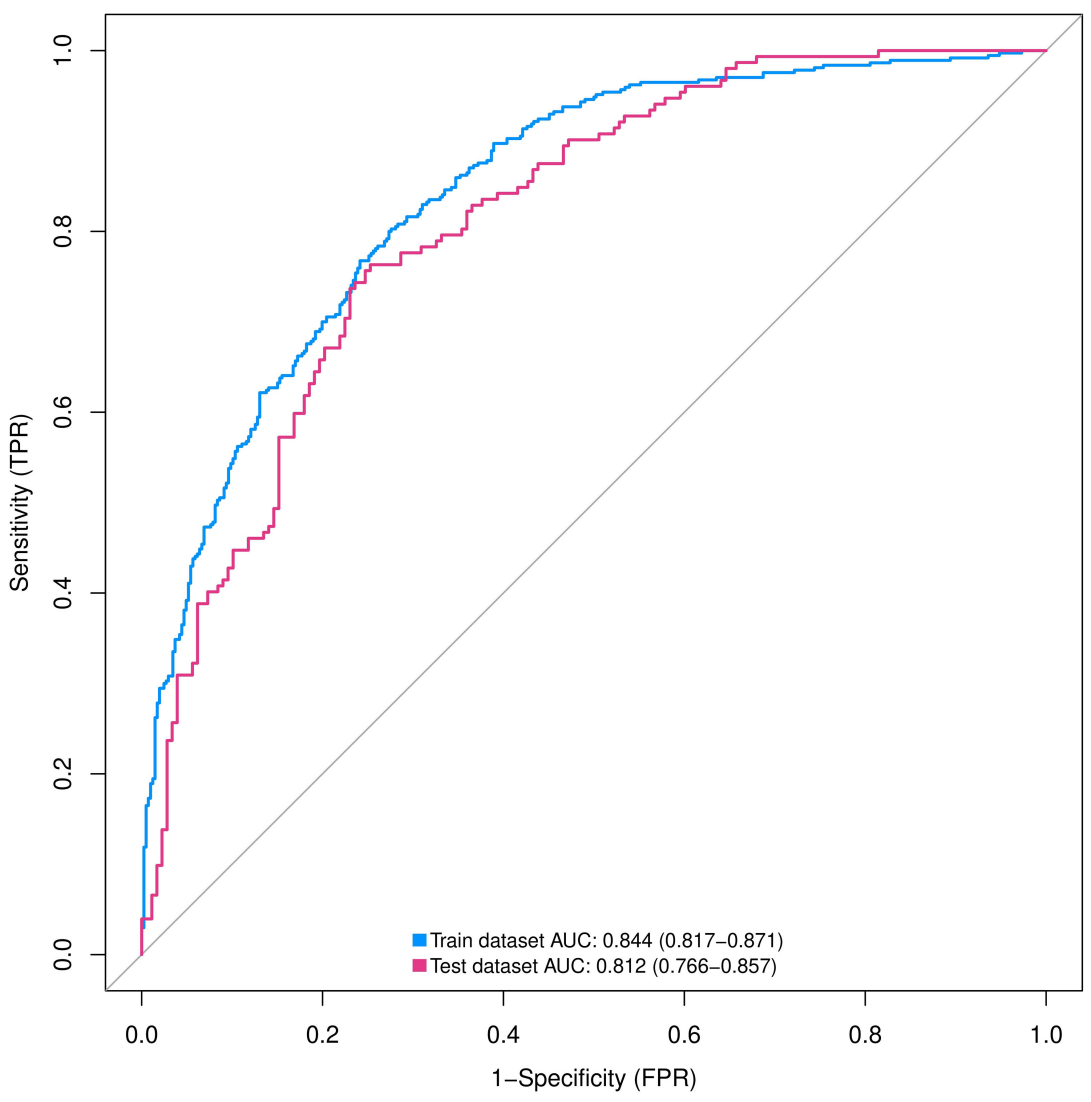
ROC curves of nomograms predicting IPA in training and validation groups. AUC, area under the ROC curve; ROC, receiver operating characteristic; SPN, solitary pulmonary nodule.

**Figure 5:**
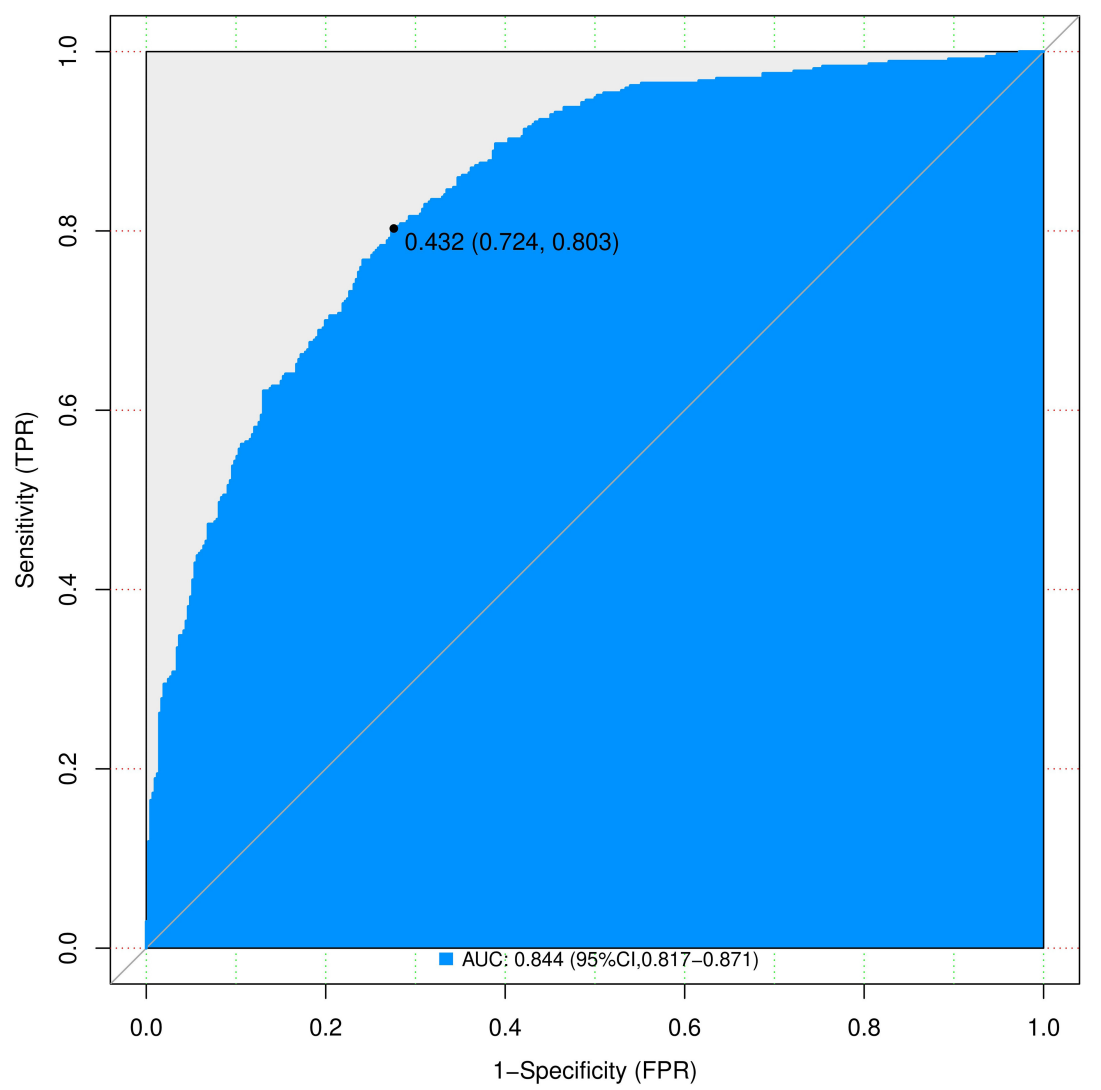
Complex ROC curves for nomograms to predicting IPA of SPN ≤ 2 cm in the training cohort. AUC, area under the ROC curve; ROC, receiver operating characteristic; SPN, solitary pulmonary nodule.

We assessed the discriminative power of the prediction model and nomogram using ROC curves. The AUCs of the ROC curve were 0.844 (95% CI, 0.817–0.871 and 0.812 (95% CI: 0.766–0.857) for the training and validation cohorts, respectively, indicating that the predictive accuracy of the nomogram was relatively good (**Figure 4**). Nevertheless, overfitting might have caused the high AUC values. The ROC curve truncation value for the training cohort was 0.432, with sensitivity and specificity of 0.803 and 0.724, respectively (**Figure 5**; **Table 5**).

**Table 5:** Results of ROC curve for training cohort.

Characteristics	Value
Threshold	0.432
Specificity	0.724
Sensitivity	0.803
Accuracy	0.762
TN	294
TP	297
FN	73
FP	112
NPR	0.801
PPV	0.726
FDR	0.274
FPR	0.276
TPR	0.803
TNR	0.724
FNR	0.197
1-specificity	0.276
1-sensitivity	0.197
1-accuracy	0.238
1-NPV	0.199
1-PPV	0.274
Precision	0.726
Recall	0.803
Youden index	1.527
Closest.topleft	0.115

TP, true positive; FP, false positive; TN, true negative; FN, false negative; TPR, true positive rate; FPR, false positive rate; TNR, true negative rate; FNR, false negative rate; PPV, positive predict value; NPR, negative predict value; FDR, false discovery rate.

**Figure 6:**
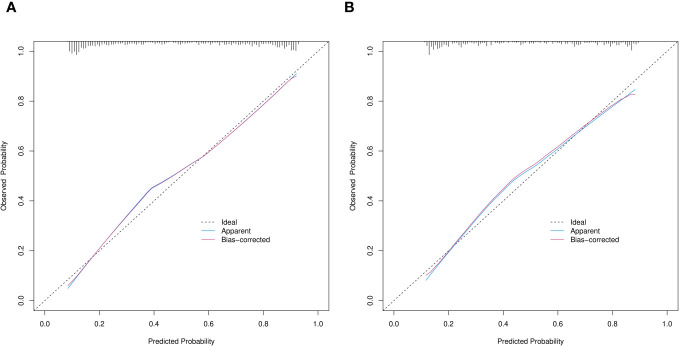
Calibration curves of prediction nomogram in training **(A)** and validation **(B)** cohorts. X and y axes respectively represent probability predicted by nomogram and actual probability of SPN ≤ 2 cm being IPA. Black dashed, blue and red solid lines, ideal, apparent (uncorrected), and deviation (corrected) curves the bootstrap method (B = 1,000 samplings). SPN, solitary pulmonary nodule.

Calibration power was evaluated using Hosmer-Lemeshow tests and calibration plots. The values for p in the Hosmer-Lemeshow test were 0.068 and 0.290 in the training and validation cohorts, respectively, indicating no significant differences between the predicted and actual probabilities. Good calibration of the predicted nomogram was also supported by calibration plots of the training (**Figure 6A**) and validation (**Figure 6B**) cohorts. The bias-corrected C-indices in the training and validation cohorts were 0.844 and 0.814, respectively.

### 3.5 Clinical utility of the predictive nomogram

**Figure 7:**
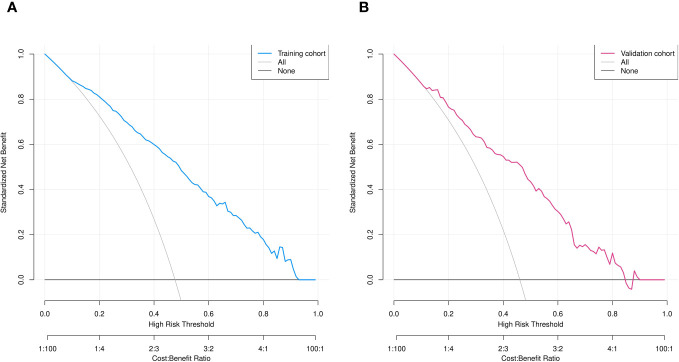
Decision curve analysis of predicted nomogram in training **(A)** and validation **(B)** cohorts. Y axis, net gain; black and grey lines, hypotheses that SPNs with diameter ≤ 2 cm are pre-IPA in nature and that SPNs ≤2 cm in diameter are IPAs. respectively. Blue **(A)** and red **(B)** lines, training and validation cohorts, respectively.

**Figure 8:**
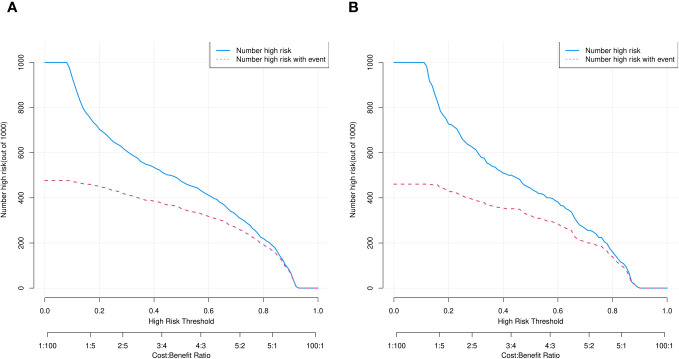
Clinical impact curves of predicted nomogram in training **(A)** and validation **(B)** cohorts. Horizontal and vertical coordinates, probability threshold and numbers of people, respectively. Numbers of individuals with SPNs judged by model as IPAs (blue line) and as IPAs and nodal true IPAs (red line) at different probability thresholds. Bottom, loss-to-benefit ratio at different probability thresholds.

We assessed the clinical utility of the nomograms using decision curve analysis. The nomograms in **Figures 7A, B**, provided greater net benefit and broader threshold probabilities for predicting the risk of IPA of SPN ≤ 2 cm in diameter in the training and validation cohorts, indicating that nomograms were clinically useful. We also created clinical impact curves (**Figure 8**) to enable surgeons to make better clinical decisions.

## 4 Discussion

Optimal management of patients with SPN is a growing clinical concern. Pathological IPA of persistent SPNs is important to assess because clinical management strategies for pre-IPA and IPA lesions are variable. We developed a clinical prediction model and visual diagnostic nomogram for individualized preoperative prediction of IPA of SPN with diameters ≤ 2 cm by retrospectively analyzing the hematological indices, imaging characteristics, and general clinical information of 776 patients in the training cohort. We identified age, CEA values, bronchial signs, lobulation, pleural adhesions, maximum tumor diameter, and CTRs as independent predictors of IPA. Our nomogram predicted patient-specific IPA probability with excellent discrimination and outstanding calibration.

Age is an important clinical factor. The capacity of cells to renew and repair epithelial damage caused by carcinogens decreases, whereas tumor malignancy increases with advancing age (25–27). Although we found that age correlated with IPA, it was the least influential factor.

Carcinoembryonic antigen is a polysaccharide protein complex involved in cell adhesion, which is usually absent or minimal in healthy adult blood and it might be linked to the poor prognosis of tumors (28). Elevated serum CEA levels are significant predictive markers of early relapse (29), progression (30), and treatment outcomes. Our findings showed that CEA can predict the IPA of SPN, which was consistent with these previous studies.

Lobular signs are more prevalent in invasive than pre-infiltrative lesions (31). Bronchial changes can predict IPA (32). These morphological features are associated with active fibroblast proliferation in adenocarcinomas and are caused by fibrous tissue contraction (33). This has been confirmed by others, suggesting that activated fibroblast proliferation in adenocarcinoma is associated with aggressive tumor growth (34). In addition, the insignificance of spiculations here might be attributable to their low abundance. Subpleural nodules or tumors in contact with the visceral pleura or linear clouding, which is vertical and intersects the visceral pleura, might result in pleural adhesion (35). Pleural adhesions are associated with tumor invasiveness and a poor prognosis (36–38). Our findings suggested that lobar, bronchial, and pleural adhesions are more likely features of invasive lung adenocarcinoma.

The size of nodules increases in parallel as lung adenocarcinoma becomes more invasive (39, 40). Our findings confirmed this. Moreover, the maximum nodule diameter was the most influential factor for IPA in the present study.

The CTR is an imaging feature of small lung adenocarcinomas and is the ratio of the diameter of solid tumors to that of the total tumor (41–43). It is an established radiological parameter used to identify pathologically noninvasive tumors on CT images (43, 44). We found that the CTR positively correlated with IPA. Thus, a higher proportion of solid components is associated with more invasive SPNs.

We used data from Qilu Hospital to develop and validate a new predictive model and clinical prediction nomogram that can help thoracic surgeons use preoperative information to assess risk of IPA in patients with SPNs. Patients with high scores underwent curative lobectomy, whereas those with low scores underwent sublobar resection. Consequently, modeling to distinguish between IPA and pre-IPA in patients with SPNs can improve their management and prognosis.

The PKUPH model was said to be better than conventional models, whereas the Mayo model was the most often used model for predicting malignant SPN. A more precise forecasting technique based on CT scans and descriptions of clinical data is the Brock model. Nevertheless, clinical indicators were not incorporated in these models. Chinese mainland populations are not a good fit for foreign prediction models. Certain prediction models integrate more complex and quantitative imaging data into their evaluations, such as tumor diameter growth rates and CT attenuation. However, due to their difficulty in obtaining, conducting, and standardising, these imaging data are rarely recognised and utilised by physicians. Unlike previous studies (45), we introduced benign tumors and combined them with the pre-IPA group. This grouping method is useful for predicting the prognosis of patients and it has value in guiding clinical decisions. This is because the possibility of benign tumors cannot be completely excluded from clinical SPNs. Moreover, we incorporated basic clinical patient information, imaging features, and hematological findings to establish a clinical prediction model with comprehensive preoperative information. The combination of preoperative clinical predictive results and rapid intraoperative pathological findings allows accurate and safe realization of nodal aggressiveness and the development of treatment strategies that are specific for individual patients. In cases where predictive modelling suggested, before surgery, that there was a high likelihood the nodule was invasive, we operated on the patient and performed a lobectomy. If preoperative predictive modelling suggested that the nodule was likely non-invasive, we performed a sublobar excision to maintain the patient’s lung function. Each patient therefore receives a customised diagnosis and course of care.

This study had several limitations. We included only patients who underwent surgical resection in our department. Those who did not undergo surgical resection were excluded, which represents selection bias. The subjectivity of radiologists might have led to different judgments of the CT images of pulmonary nodules. Our model was limited by the retrospective design of study. Our data were derived from a single center with a relatively small sample size. The predictive model has only been validated internally, and further validation involving multiple centers and sufficient samples are needed. Although the validation of the model showed good discriminatory and calibration capabilities, the generalizability of nomograms to new patient populations remains a major issue. However, the nomogram requires further external validation.

## 5 Conclusions

We developed and validated a novel and easy-to-use nomogram for predicting the risk of IPA in patients with SPN ≤ 2 cm in diameter. With excellent differentiation and calibration, clinicians and surgeons can accurately develop specific treatment strategies for each patient.

## Data availability statement

The raw data supporting the conclusions of this article will be made available by the authors, without undue reservation.

## Ethics statement

The Ethics Committee of Qilu Hospital, Shandong University approved this single-center study (registration number: KYLL-202008-023-1). The studies were conducted in accordance with the local legislation and institutional requirements. Written informed consent for participation was not required from the participants or the participants’ legal guardians/next of kin in accordance with the national legislation and institutional requirements.

## Author contributions

MX: Writing – review & editing, Writing – original draft, Visualization, Validation, Supervision, Software, Resources, Project administration, Methodology, Investigation, Formal analysis, Data curation, Conceptualization. RL: Writing – original draft, Visualization, Supervision, Software, Resources, Project administration, Methodology, Investigation, Formal analysis. JL: Writing – review & editing, Resources, Project administration, Methodology, Investigation. ML: Writing – original draft, Visualization, Validation, Supervision, Software, Resources, Project administration, Methodology, Investigation, Formal analysis, Data curation, Conceptualization. ZL: Writing – review & editing, Funding acquisition, Formal analysis, Data curation, Conceptualization. HZ: Writing – review & editing, Resources, Project administration, Methodology, Investigation. HT: Writing – review & editing, Writing – original draft, Visualization, Validation, Supervision, Software, Resources, Project administration, Methodology, Investigation, Funding acquisition, Formal analysis, Data curation, Conceptualization.
