# Supplementary figures and images for: Nomogram for predicting invasive lung adenocarcinoma in small solitary pulmonary nodules
Source: Front Oncol. 2024 Jul 1;14:1334504. doi: 10.3389/fonc.2024.1334504 (PMC11246902; doi:10.3389/fonc.2024.1334504)

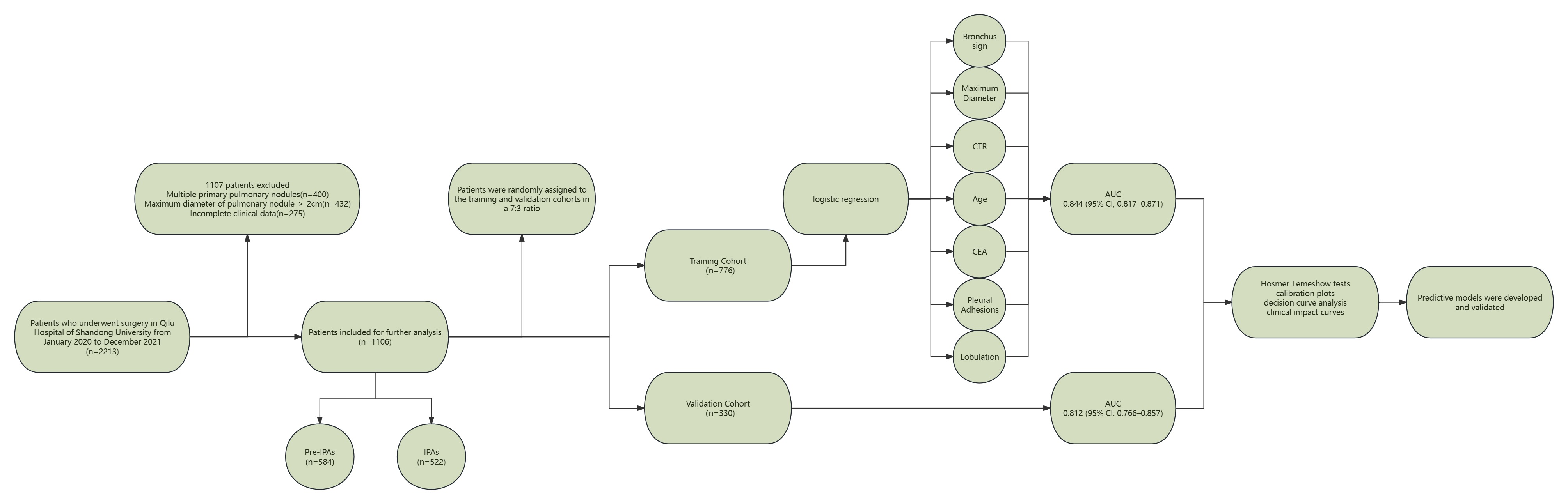

Supplement: Supplementary file 2 [file Image_1.jpeg]
